# Multimodal Diagnosis of Canine Leishmaniosis in a Dog Relocated from Italy to Romania: A Case Report

**DOI:** 10.3390/pathogens15101018

**Published:** 2026-09-26

**Authors:** Bianca-Lavinia Andronic, Larisa-Maria Ivănescu, Raluca Mîndru, Gabriela-Victoria Apopei, Liviu Miron

**Affiliations:** Department of Clinics, Faculty of Veterinary Medicine, “Ion Ionescu de la Brad” Iasi University of Life Sciences, 8 Mihail Sadoveanu Alley, 700490 Iași, Romania; lavinia.andronic@iuls.ro (B.-L.A.); raluca.mindru@iuls.ro (R.M.); gabriela.martinescu@iuls.ro (G.-V.A.); liviu.miron@iuls.ro (L.M.)

**Keywords:** canine leishmaniosis, parasitic infection, *Leishmania*, conjunctival swab, molecular diagnosis, serology

## Abstract

Canine leishmaniosis is a chronic vector-borne parasitic zoonosis caused predominantly by *Leishmania infantum* in Europe, with dogs being the main domestic reservoir. Diagnosis may be challenging in countries where the disease is less frequently suspected, particularly in relocated dogs from endemic regions. This case report describes an integrated diagnostic approach for canine leishmaniosis in a six-year-old male Cane Corso dog relocated from Tuscany, Italy, to north-eastern Romania. The dog was referred for progressive weight loss and poor general condition. Clinical examination revealed cachexia, muscle wasting, onychogryphosis, severe pustular pododermatitis, ulcerative lesions of the plantar pads, auricular erosions with fine scaling, nasal planum hyperkeratosis and perianal erythema. Laboratory abnormalities included moderate anaemia, marked hyperproteinaemia, hyperglobulinaemia, hypoalbuminaemia and a markedly decreased albumin-to-globulin ratio. Cytological examination of a May–Grünwald–Giemsa-stained sternal bone-marrow aspirate revealed intracellular amastigote-like forms compatible with *Leishmania* spp. Anti-*Leishmania* antibodies were detected using three complementary serological methods: a point-of-care rapid antibody test, IFAT and ELISA. Molecular detection of *Leishmania* spp. DNA was achieved by real-time PCR performed on DNA extracted from a conjunctival swab using two independent protocols, including a TaqMan-based assay targeting *Leishmania* spp. minicircle kDNA. Treatment consisted of meglumine antimoniate administered in combination with oral allopurinol, which was subsequently continued as long-term maintenance therapy, together with supportive treatment and ectoparasite prophylaxis. This case underscores the importance of considering canine leishmaniosis in relocated dogs with compatible clinical findings and of integrating epidemiological history with complementary diagnostic evidence, particularly in regions where the disease is infrequently encountered. From a One Health perspective, the case also highlights the relevance of surveillance in dogs relocated from endemic areas to regions where competent vectors are present and autochthonous transmission has been documented.

## 1. Introduction

Canine leishmaniosis (CanL), caused in Europe predominantly by *Leishmania infantum* [1], is a vector-borne zoonotic disease transmitted by phlebotomine sand flies [2]. Dogs represent the main domestic reservoir of the parasite and play an important role in the epidemiology of zoonotic transmission [3]. The infection may remain subclinical for prolonged periods or progress to systemic disease, depending on the immune system response of the host [4]. Clinical findings are highly variable and may include weight loss, dermatological lesions, lymphadenopathy, anaemia, hyperglobulinaemia, hypoalbuminaemia and renal involvement [5,6]. This clinical heterogeneity can complicate the diagnosis, especially in regions where CanL is rarely encountered and therefore may not be initially included among the main differential diagnoses.

The diagnosis of CanL is based on the integration of clinical and clinicopathological findings with etiological, serological and molecular evidence. However, individual diagnostic methods have inherent limitations, and discordant results may occur depending on the disease stage, immune response, parasite burden and the biological sample analysed [5,7]. In clinical cases, concordance among findings obtained using different diagnostic techniques may strengthen the interpretation of infection status, particularly when the disease is not initially suspected.

Romania has historically been considered a low-risk and non-endemic country for leishmaniosis. Nevertheless, autochthonous canine infections and subclinical *L. infantum* circulation have been documented [8,9], while subsequent surveys have provided additional serological and molecular evidence of infection in Romanian dog populations [10,11]. Phlebotomine sand flies, including species involved in *L. infantum* transmission, have also been recorded in Romania [12]. Several clinical cases have been diagnosed in dogs originating from endemic Mediterranean countries and subsequently relocated to Romania [13,14,15]. Increased movement of companion animals between Romania and Mediterranean endemic countries further complicates the epidemiological context. Consequently, a history of residence or travel in Mediterranean countries remains highly relevant, although it cannot by itself establish the geographical origin of infection.

The present case report describes canine leishmaniosis in a six-year-old Cane Corso relocated from Tuscany, Italy, to north-eastern Romania and illustrates the practical relevance of integrating epidemiological history with complementary diagnostic findings when evaluating dogs presenting in regions where canine leishmaniosis may not be routinely suspected.

## 2. Materials and Methods

In July 2024, a six-year-old intact male Cane Corso weighing 42 kg was referred to the Faculty of Veterinary Medicine in Iași, Romania. The dog had lived in Tuscany, Italy during its first five years of life and had resided in north-eastern Romania for approximately one year before presentation. The owners noted progressive weight loss over several months, with an evident decline after the dog had spent four months under another person’s care. The medical history included bilateral hip dysplasia and previous surgery of the right hind limb. No further international travel was reported after relocation to Romania. The dog had not previously been tested for *Leishmania* infection while living in Italy. External antiparasitic prophylaxis had been administered inconsistently, and no regular sand-fly specific repellent prophylaxis was reported. Treatment with sarolaner was documented approximately one month before presentation. Specific seasonal exposure to phlebotomine sand flies could not be retrospectively established. Diagnostic investigations were undertaken during the July 2024 referral, and specific treatment was initiated following completion of the diagnostic work-up. A clinical re-evaluation was recommended for September 2024.

A complete physical and dermatological examination was performed. Blood was collected for a complete blood count, serum biochemistry and anti-*Leishmania* antibody testing. Abdominal ultrasonography and parasitological examination of a skin scraping were also performed. A sternal bone-marrow aspirate was collected, smeared onto a glass slide, stained with May–Grünwald–Giemsa and examined by light microscopy using a ×100 oil-immersion objective. A conjunctival swab was also collected for molecular detection of *Leishmania* spp. DNA by real-time PCR.

Serological testing was performed sequentially using three different methods. First, a point-of-care rapid antibody test was used as an initial screening assay. Subsequently, an indirect immunofluorescence antibody test (IFAT) was performed, and an enzyme-linked immunosorbent assay (ELISA) was subsequently performed as an additional serological assay.

The initial screening was performed using the SNAP *Leishmania* test (IDEXX Laboratories, Inc., Westbrook, ME, USA), according to the manufacturer’s instructions. The result was read after 8 min. The test was considered positive when both the control spot and the sample spot developed.

IFAT was performed using a commercial indirect immunofluorescence kit for the detection of anti-*Leishmania* antibodies in dog serum, based on *Leishmania infantum* antigen-coated slides (Anti-*Leishmania* Antibodies, BioSystems S.A., Barcelona, Spain), according to the manufacturer’s instructions. The serum sample was screened at a 1:80 dilution in phosphate-buffered saline. Positive and negative controls supplied with the kit were included in the assay. After incubation and washing, the slides were treated with fluorescein-labelled anti-dog IgG conjugate and examined using a fluorescence microscope. The sample was considered positive when specific green fluorescence of *L. infantum* promastigotes was observed.

ELISA was subsequently performed using a commercial indirect ELISA kit for the detection of anti-*Leishmania infantum* antibodies in dog serum (ID Screen^®^ Leishmaniasis Indirect, ID.vet/Innovative Diagnostics, Grabels, France), according to the manufacturer’s instructions. Serum and control samples were processed in a 96-well antigen-coated microplate format. After incubation, washing, addition of conjugate, substrate and stop solution, optical density was read at 450 nm. The assay was considered valid when the mean optical density of the positive control was greater than 0.350 and the positive-to-negative control ratio was greater than 3. The sample-to-positive control percentage (S/P%) was calculated according to the manufacturer’s formula. Samples with S/P% ≤ 40% were considered negative, samples with 40% < S/P% < 50% were considered doubtful, and samples with S/P% ≥ 50% were considered positive.

A conjunctival swab was collected for molecular detection of *Leishmania* DNA. Exfoliative conjunctival material was obtained using a sterile swab by gently rubbing the conjunctival surface and the swab was subsequently processed for DNA extraction. Genomic DNA was extracted using the PureLink™ Genomic DNA kit (Thermo Fisher Scientific, Waltham, MA, USA), according to the manufacturer’s instructions. The final elution was performed in 50 µL of elution buffer.

Real-time PCR was performed using two complementary protocols. First, a commercially available probe-based real-time PCR assay targeting the 70 kDa heat shock protein (HSP70) gene for the detection of *Leishmania* spp. DNA (Omnigen, Bucharest, Romania) was performed. The amplification and result-validation framework followed the real-time PCR workflow previously described by Turcitu [16]. The reaction was prepared in a final volume of 20 µL, consisting of 5 µL of real-time PCR master mix (QuantiNova Pathogen + IC Kit, Qiagen, Hilden, Germany), 7 µL of extracted DNA and 8 µL of the commercial primer/probe mixture. The primer and probe sequences and their individual concentrations are proprietary components of the commercial assay and were not disclosed by the supplier.

Amplification was carried out on a CFX96 Real-Time PCR Detection System (Bio-Rad Laboratories, Hercules, CA, USA). The thermocycling programme consisted of an initial denaturation step at 95 °C for 2 min, followed by 40 cycles of 95 °C for 15 s and 62 °C for 1 min. Fluorescence signals were analysed using CFX Manager Software version 3.1 (Bio-Rad Laboratories, Hercules, CA, USA).

The *Leishmania*-specific target was detected in the FAM channel. Result validation included an endogenous internal control detected in the Cy5 channel, targeting a mammalian housekeeping gene to assess sample cellularity and extraction/amplification adequacy, and an exogenous internal control detected in the HEX channel to monitor potential amplification inhibition. A positive amplification control and a no-template control (NTC) were included in the run. Samples showing a specific amplification curve in the FAM channel with a Cq value < 40, together with valid internal controls and absence of target amplification in the NTC, were considered positive.

Secondly, the conjunctival swab DNA was tested using a singleplex TaqMan-based real-time PCR assay targeting a 120 bp fragment of *Leishmania* spp. minicircle kinetoplast DNA (kDNA), adapted from Sales et al. [17] and based on the assay previously described by Francino et al. [18]. The assay used the primers LEISH-1 forward, 5′-AACTTTTCTGGTCCTCCGGGTAG-3′, LEISH-2 reverse, 5′-ACCCCCAGTTTCCCGCC-3′, and the TaqMan-MGB probe FAM-AAAAATGGGTGCAGAAAT-NFQ-MGB. The reaction mixture had a final volume of 15 µL, and consisted of 7.5 µL TaqMan Fast Advanced Master Mix (Thermo Fisher Scientific, Waltham, MA, USA), 1.35 µL of each primer, 0.3 µL probe, 2.5 µL nuclease-free water and 2 µL DNA template. The final concentrations were 900 nM for each primer and 200 nM for the probe. Thermal cycling conditions were: 20 s at 95 °C, followed by 35 cycles of 1 s at 95 °C and 20 s at 60 °C. Reactions were performed in duplicate on a CFX96 Real-Time PCR Detection System (Bio-Rad Laboratories, Hercules, CA, USA), using automatic baseline and threshold settings. DNA extracted from a bone-marrow aspirate obtained from a dog previously confirmed positive for canine leishmaniosis was used as a positive control, and a no-template control was included in the run.

## 3. Results

Clinical examination revealed cachexia, moderate muscle wasting and poor general condition. Dermatological findings included severe pustular pododermatitis affecting all four limbs, ulcerative lesions on the plantar pads of the right forelimb, onychogryphosis, bilateral erosions of the auricular pinnae with fine scaling, hyperkeratosis of the nasal planum and perianal erythema (Figure 1). Abdominal ultrasonography revealed no clinically relevant abnormalities, while parasitological examination of the skin scraping was negative for ectoparasites.

The complete blood count revealed moderate anaemia (RBC 4.77 × 10^12^/L; reference interval [RI]: 5.5–8.5 × 10^12^/L), with haemoglobin of 9.3 g/dL (RI: 12–18 g/dL) and haematocrit of 28.74% (RI: 37–55%). Serum biochemistry showed marked hyperproteinaemia, with total proteins of 10.1 g/dL (RI: 5.4–8.2 g/dL), associated with hyperglobulinaemia of 8.2 g/dL (RI: 2.3–5.2 g/dL), hypoalbuminaemia of 1.9 g/dL (RI: 2.5–4.4 g/dL) and a markedly decreased albumin-to-globulin ratio of 0.23 (Table 1). Renal biochemical parameters showed no evidence of azotaemia, with serum creatinine of 0.6 mg/dL (RI: 0.3–1.4 mg/dL) and BUN of 6 mg/dL (RI: 7–25 mg/dL).

Cytological examination of the May–Grünwald–Giemsa-stained sternal bone-marrow aspirate revealed intracellular amastigote-like forms compatible with *Leishmania* spp. (Figure 2).

The point-of-care SNAP *Leishmania* test was positive, indicating the presence of anti-*Leishmania* antibodies (Figure 3). IFAT was also positive at the 1:80 screening dilution, with specific green fluorescence observed on *L. infantum* promastigotes (Figure 4). Serial endpoint titration was not performed; therefore, an endpoint antibody titre was not available. ELISA was also positive, with an S/P value of 183.25%, above the manufacturer’s positivity threshold of 50%.

*Leishmania* spp. DNA was detected in the conjunctival swab using two real-time PCR protocols (Figure 5).

The HSP70-targeting real-time PCR assay detected *Leishmania* spp. DNA in the conjunctival swab sample, with specific amplification in the FAM channel at Cq 35.22, which was within the assay positivity criterion (Cq < 40). The positive amplification control amplified at Cq 17.42, whereas no target amplification was observed in the NTC. The endogenous internal control detected in the Cy5 channel amplified appropriately, supporting adequate sample cellularity and extraction/amplification performance, while the exogenous internal control detected in the HEX channel indicated the absence of detectable PCR inhibition.

The molecular result was further confirmed using a second singleplex TaqMan-based real-time PCR assay targeting *Leishmania* spp. minicircle kDNA, adapted from Sales et al. [17]. In this assay, amplification was detected only in the FAM channel. The positive control amplified in duplicate, with Cq values of 27.78 and 28.16, while the conjunctival swab also amplified in duplicate, with Cq values of 29.71 and 29.74. No amplification was observed in the no-template controls.

Because the two real-time PCR assays differed in molecular target, reaction design and analytical characteristics, their Cq values were reported descriptively and were not directly compared as indicators of parasite burden.

Following diagnosis, treatment was initiated with meglumine antimoniate at 100 mg/kg body weight subcutaneously once daily for 30 days, together with allopurinol at 10 mg/kg body weight orally twice daily. Allopurinol was continued long-term at the same dose and frequency. Supportive treatment and external ectoparasite prophylaxis with repellent activity against phlebotomine sand flies were also recommended.

## 4. Discussion

The clinical and clinicopathological findings in this dog were highly compatible with canine leishmaniosis. Weight loss, poor body condition and dermatological lesions are commonly reported in clinically affected dogs, while anaemia, hyperproteinaemia, hyperglobulinaemia and hypoalbuminaemia are well-recognised laboratory abnormalities [5,6]. Hyperglobulinaemia reflects marked humoral immune stimulation, whereas hypoalbuminaemia may be associated with chronic inflammation, renal involvement or protein loss [5,6]. The markedly decreased albumin-to-globulin ratio observed in this dog was therefore consistent with a chronic inflammatory or infectious process. Complete clinical staging according to the LeishVet system [5] could not be reliably performed because urinalysis and the urinary protein-to-creatinine ratio were not available. Although serum creatinine was within the reference interval and no azotaemia was detected, clinically relevant proteinuria could not be excluded. Therefore, a definitive LeishVet stage was not assigned.

Cytological examination of the sternal bone-marrow aspirate revealed intracellular amastigote-like forms compatible with *Leishmania* spp., providing direct parasitological evidence consistent with infection. Although cytology may have limited sensitivity depending on parasite load and sample quality [5], this finding was concordant with the serological and molecular results. Serological testing was positive by SNAP *Leishmania*, IFAT and ELISA. IFAT was positive at the 1:80 screening dilution; however, serial endpoint titration was not performed and an endpoint antibody titre was therefore not available. The ELISA S/P value was clearly above the manufacturer’s positivity threshold and reflected a strong relative antibody signal, but should not be interpreted as a measure of parasite burden.

Molecular detection from the conjunctival swab provided additional support for the diagnosis. Conjunctival swabs are non-invasive samples that have been proposed as useful for molecular detection of *Leishmania* infection in dogs, particularly when invasive sampling is undesirable [19,20]. In the present case, *Leishmania* spp. DNA was detected using two real-time PCR approaches, including a kDNA-targeting TaqMan assay that produced reproducible amplification in duplicate. Nevertheless, neither molecular assay was designed for species-level discrimination. Therefore, the molecular findings demonstrate the presence of *Leishmania* spp. DNA but do not constitute molecular identification of *L. infantum*. Considering the predominance of *L. infantum* as the agent of canine leishmaniosis in Europe [1] and its endemic occurrence in Italy [21], this species remains the most epidemiologically plausible aetiological agent in this dog, although species-level confirmation was not performed.

The epidemiological history further supports infection acquired before relocation as the most plausible scenario. The dog had spent the first five years of its life in Tuscany, Italy, where canine leishmaniosis is endemic [22], had not previously been tested for *Leishmania* infection, and had received external antiparasitic prophylaxis inconsistently, without regular sand-fly specific repellent protection. Moreover, canine leishmaniosis may remain subclinical for prolonged periods before clinical signs become apparent [4,5]. Although local acquisition in Romania cannot be completely excluded, the prolonged residence in an endemic area makes infection acquired in Italy more epidemiologically plausible. The exact geographical origin of infection cannot, however, be established with certainty because autochthonous canine infections and competent phlebotomine sand flies have been documented in Romania [8,9,12].

Treatment consisted of meglumine antimoniate in combination with oral allopurinol, followed by long-term allopurinol therapy, together with supportive treatment. This combination represents one of the recommended first-line therapeutic approaches for canine leishmaniosis [5,6]. Regular external ectoparasite prophylaxis with repellent activity against phlebotomine sand flies was also recommended because infected dogs may act as reservoirs for *Leishmania* parasites [3] and prevention of sand fly exposure remains an important component of disease control [23,24]. A clinical re-evaluation was recommended; however, the dog did not return to our institution because subsequent clinical management was continued by the attending veterinarian in the owner’s county of residence. The owner subsequently reported improvement in the dog’s general condition and clinical signs, but no objective follow-up examination, complete blood count, serum biochemistry or serological data were available.

Canine leishmaniosis, including cases diagnosed in dogs originating from endemic Mediterranean countries, has previously been reported in Romania [13,14,15]. Therefore, the occurrence of the disease in a relocated dog is not itself novel. The relevance of the present case lies instead in the concordant clinical, clinicopathological, cytological, serological and molecular evidence documented in a dog presented in north-eastern Romania, where canine leishmaniosis may not routinely be considered among the initial differential diagnoses. The case also illustrates the importance of integrating epidemiological history with diagnostic findings when evaluating dogs relocated from endemic areas.

Other vector-borne diseases and chronic inflammatory or immune-mediated disorders were considered relevant differential diagnoses [5,6]. However, systematic screening for other vector-borne infections was not performed; therefore, concomitant infections could not be completely excluded [25,26]. Nevertheless, the overall concordance among the diagnostic findings strongly supported canine leishmaniosis as the principal diagnosis.

## 5. Conclusions

Canine leishmaniosis should be considered in dogs with compatible clinical and clinicopathological findings and a history of residence in or travel to endemic areas, even when they are presented in regions where the disease is infrequently encountered. In the present case, concordant cytological, serological and molecular findings provided strong support for the diagnosis. The case highlights the practical importance of combining epidemiological history with appropriate diagnostic investigation when evaluating relocated dogs in areas where canine leishmaniosis may not be routinely suspected.

## Figures and Tables

**Figure 1:**
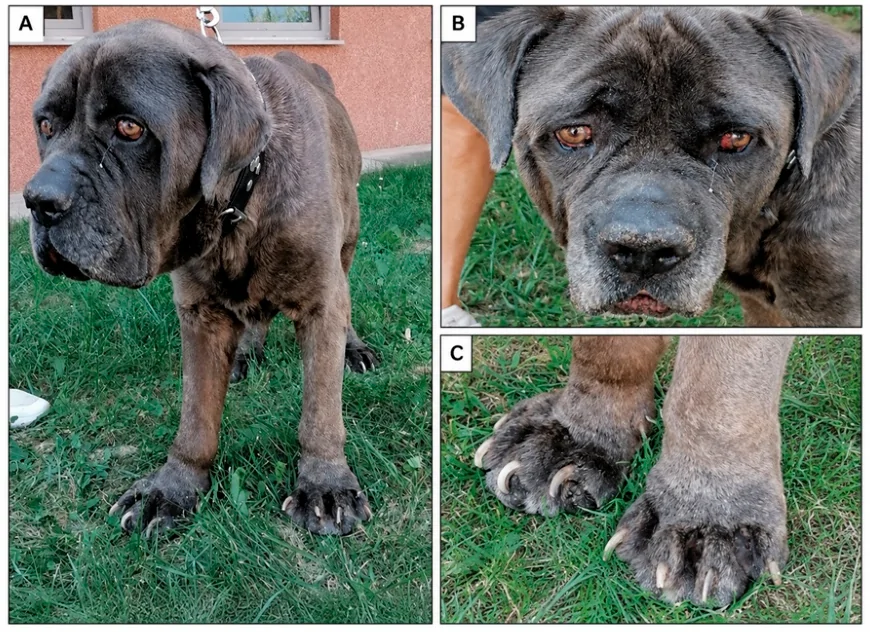
Clinical appearance of the dog at presentation. (**A**) Cachexia and generalised muscle wasting. (**B**) Scaling, hyperkeratosis of the nasal planum and ocular discharge. (**C**) Onychogryphosis.

**Figure 2:**
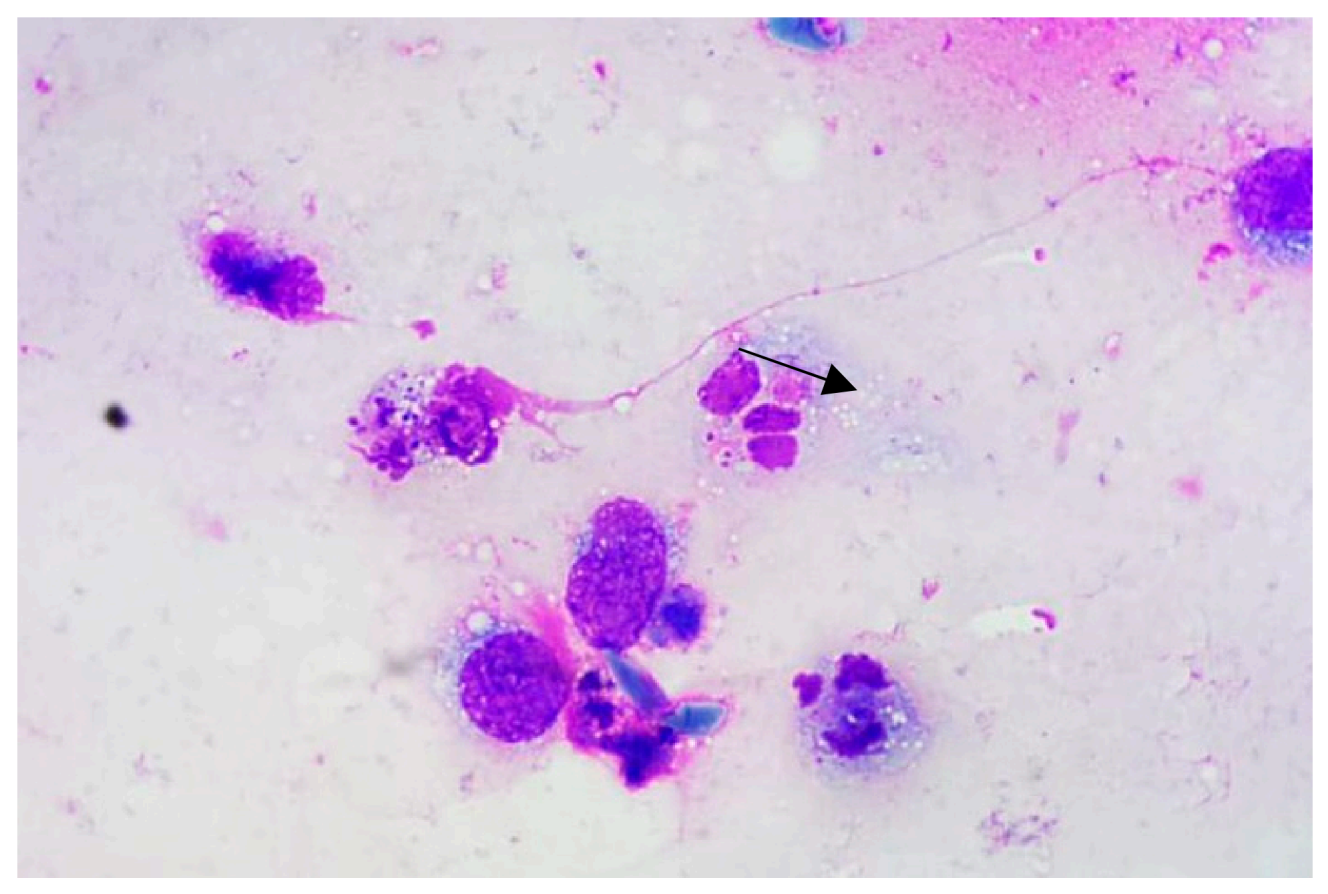
Amastigote-like forms (arrow) compatible with *Leishmania* spp. in sternal bone-marrow aspirate from the dog, May–Grünwald–Giemsa stain (×100).

**Figure 3:**
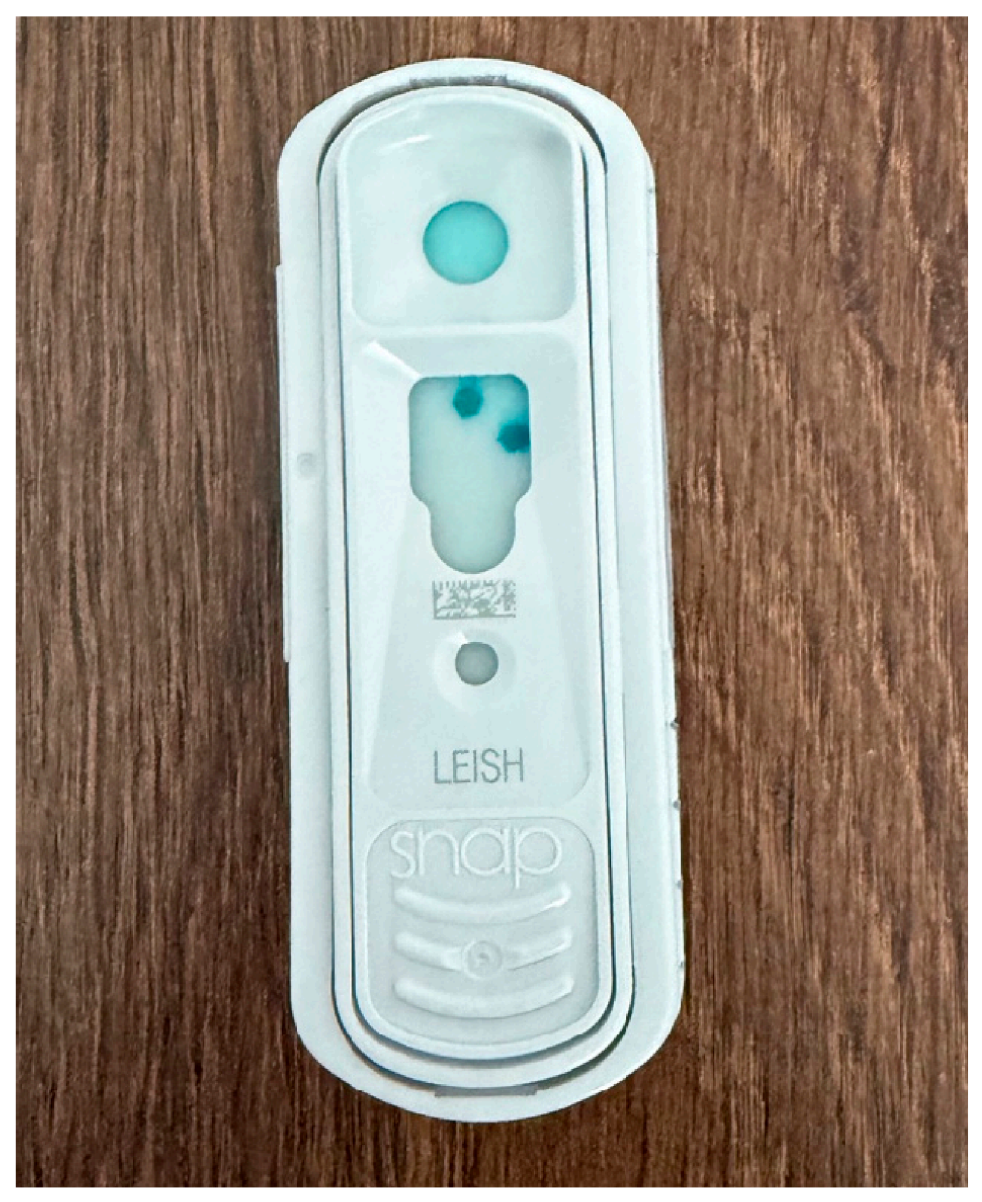
Positive SNAP *Leishmania* test, showing development of both the control and sample spots.

**Figure 4:**
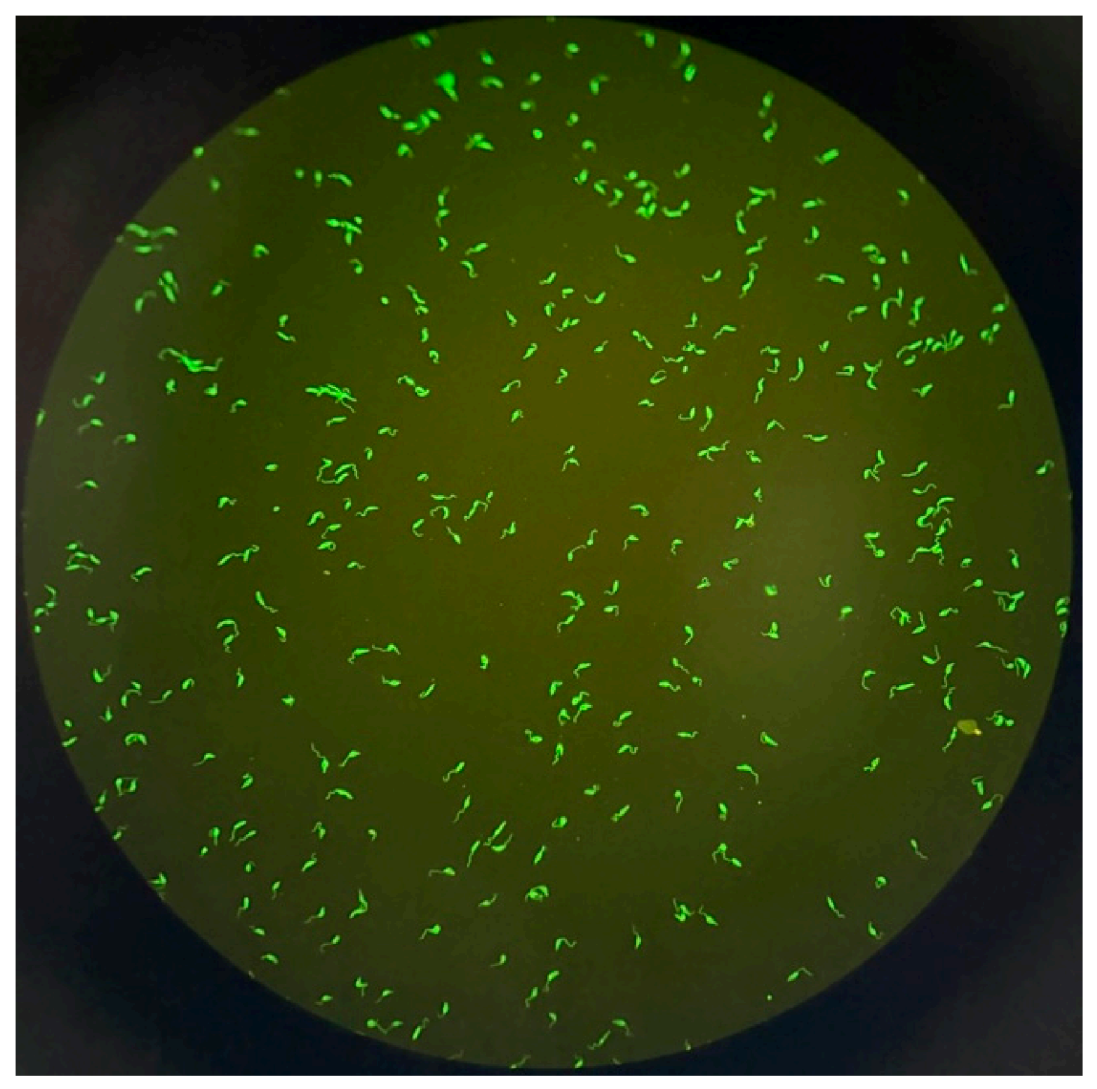
Positive indirect immunofluorescence antibody test (IFAT) at the 1:80 screening dilution, showing specific green fluorescence of *Leishmania infantum* promastigotes.

**Figure 5:**
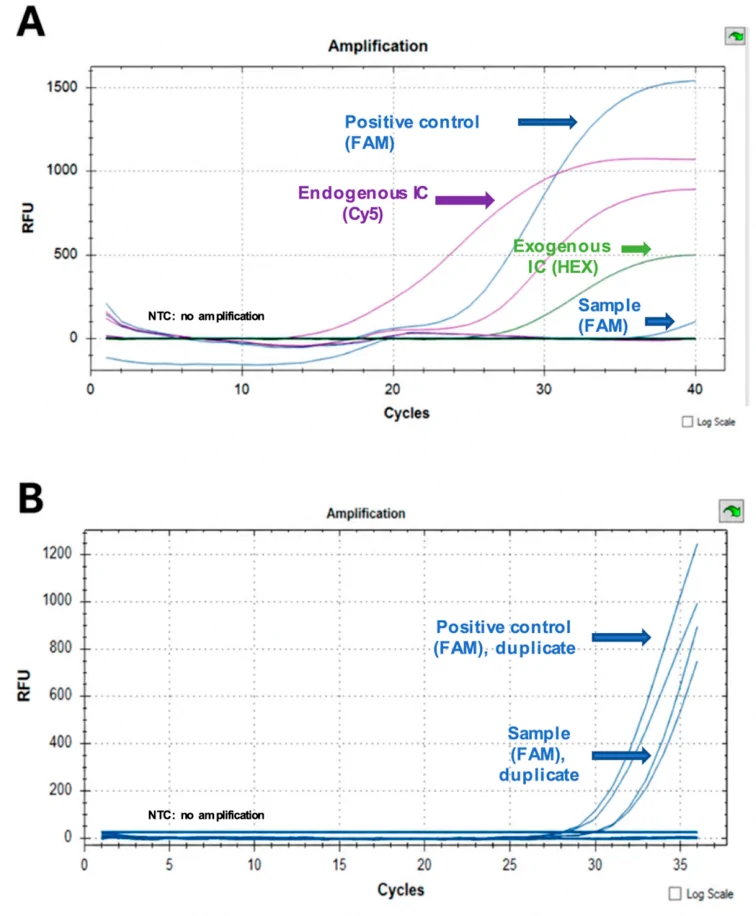
Real-time PCR detection of *Leishmania* spp. DNA from the conjunctival swab. (**A**) Commercial HSP70-targeting probe-based real-time PCR assay (Omnigen, Bucharest, Romania). The blue amplification curves correspond to the FAM signals of the positive amplification control and the clinical conjunctival swab sample; the purple curve corresponds to the endogenous internal control in the Cy5 channel, and the green curve corresponds to the exogenous internal control in the HEX channel. No amplification was observed in the no-template control (NTC). (**B**) Singleplex TaqMan-based assay targeting *Leishmania* spp. minicircle kDNA. The blue amplification curves correspond to the FAM signals of the positive control and clinical conjunctival swab sample, each tested in duplicate. No amplification was observed in the NTC.

**Table 1:** Main haematological and serum biochemical abnormalities recorded in the dog at presentation.

Parameter	Result	Reference Interval
RBC	4.77 × 10^12^/L	5.5–8.5 × 10^12^/L
Haemoglobin	9.3 g/dL	12–18 g/dL
Haematocrit	28.74%	37–55%
Total proteins	10.1 g/dL	5.4–8.2 g/dL
Albumin	1.9 g/dL	2.5–4.4 g/dL
Globulins	8.2 g/dL	2.3–5.2 g/dL
Albumin/globulin ratio	0.23	N/A

Abbreviations: RBC, red blood cell count; N/A, not available. Reference intervals were those provided by the clinical laboratory.

## Data Availability

The data presented in this study are available on request from the corresponding author.

## Abbreviations

The following abbreviations are used in this manuscript:

CanL	Canine leishmaniosis
BUN	Blood urea nitrogen
Cq	Quantification cycle
Cy5	Cyanine 5
DNA	Deoxyribonucleic acid
ELISA	Enzyme-linked immunosorbent assay
FAM	6-carboxyfluorescein
HEX	Hexachlorofluorescein
IFAT	Indirect immunofluorescence antibody test
kDNA	Kinetoplast DNA
MGB	Minor groove binder
NFQ	Non-fluorescent quencher
PCR	Polymerase chain reaction
RBC	Red blood cell count
RI	Reference interval
S/P	Sample-to-positive control percentage

## References

[1] Maia C., Conceição C., Pereira A., Rocha R., Ortuño M., Muñoz C., Jumakanova Z., Pérez-Cutillas P., Özbel Y., Töz S. (2023). The Estimated Distribution of Autochthonous Leishmaniasis by *Leishmania infantum* in Europe in 2005–2020. PLoS Neglected Trop. Dis..

[2] Ready P.D. (2010). Leishmaniasis Emergence in Europe. Eurosurveillance.

[3] Baneth G., Koutinas A.F., Solano-Gallego L., Bourdeau P., Ferrer L. (2008). Canine Leishmaniosis—New Concepts and Insights on an Expanding Zoonosis: Part One. Trends Parasitol..

[4] Ivănescu L., Andronic B.L., Grigore-Hristodorescu S., Martinescu G.V., Mîndru R., Miron L. (2023). The Immune Response in Canine and Human Leishmaniasis and How This Influences the Diagnosis- a Review and Assessment of Recent Research. Front. Cell. Infect. Microbiol..

[5] Solano-Gallego L., Miró G., Koutinas A., Cardoso L., Pennisi M.G., Ferrer L., Bourdeau P., Oliva G., Baneth G. (2011). LeishVet Guidelines for the Practical Management of Canine Leishmaniosis. Parasites Vectors.

[6] Saridomichelakis M.N., Baneth G., Colombo S., Dantas-Torres F., Ferrer L., Fondati A., Miró G., Ordeix L., Otranto D., Noli C. (2025). World Association for Veterinary Dermatology Consensus Statement for Diagnosis, and Evidence-Based Clinical Practice Guidelines for Treatment and Prevention of Canine Leishmaniosis. Vet. Dermatol..

[7] Solano-Gallego L., Morell P., Arboix M., Alberola J., Ferrer L. (2001). Prevalence of *Leishmania Infantum* Infection in Dogs Living in an Area of Canine Leishmaniasis Endemicity Using PCR on Several Tissues and Serology. J. Clin. Microbiol..

[8] Mircean V., Dumitrache M.O., Mircean M., Bolfa P., Györke A., Mihalca A.D. (2014). Autochthonous Canine Leishmaniasis in Romania: Neglected or (Re)Emerging?. Parasites Vectors.

[9] Dumitrache M.O., Nachum-Biala Y., Gilad M., Mircean V., Cazan C.D., Mihalca A.D., Baneth G. (2016). The Quest for Canine Leishmaniasis in Romania: The Presence of an Autochthonous Focus with Subclinical Infections in an Area Where Disease Occurred. Parasites Vectors.

[10] Cîmpan A.A., Diakou A., Papadopoulos E., Miron L.D. (2019). Serological study of exposure to *Leishmania* in dogs living in shelters, in South-East Romania. Rev. Rom. Med. Vet..

[11] Cazan C.D., Ionică A.M., Matei I.A., D’Amico G., Muñoz C., Berriatua E., Dumitrache M.O. (2020). Detection of *Leishmania infantum* DNA and Antibodies against *Anaplasma* Spp., *Borrelia burgdorferi* s.l. and *Ehrlichia canis* in a Dog Kennel in South-Central Romania. Acta Vet. Scand..

[12] Cazan C.D., Păstrav I.R., Ionică A.M., Oguz G., Erisoz Kasap O., Dvorak V., Halada P., Dumitrache M.O., Volf P., Alten B. (2019). Updates on the Distribution and Diversity of Sand Flies (Diptera: Psychodidae) in Romania. Parasites Vectors.

[13] Pavel G., Timofte D., Mocanu D., Malancus R., Solcan C. (2017). Imported Leishmaniasis in a Dog in a Sandfly-Populated Area in Northeastern Romania. J. Vet. Diagn. Investig..

[14] Tanase O.I., Daraban C., Velescu E., Boghean D., Bocaneti-Daraban F. (2018). Symptomatic Leishmaniasis in an Italian Segugio Dog from Northeastern Romania: A Case Report. Iran. J. Parasitol..

[15] Toma G.C., Taulescu M., Mircean V., Ionică A.M., Cora R., Cătoi C., Dumitrache M.O. (2018). Imported Canine Leishmaniasis in Romania: A Case Report. Bull. Univ. Agric. Sci. Vet. Med. Cluj-Napoca Vet. Med..

[16] Turcitu M.A. (2024). Universal workflow for pathogen detection using Real Time (RT)PCR. Rom. J. Vet. Med. Pharm..

[17] Sales K.G.D.S., Miranda D.E.D.O., Paiva M.H.S., Figueredo L.A., Otranto D., Dantas-Torres F. (2020). Fast Multiplex Real-Time PCR Assay for Simultaneous Detection of Dog and Human Blood and *Leishmania* Parasites in Sand Flies. Parasites Vectors.

[18] Francino O., Altet L., Sánchez-Robert E., Rodriguez A., Solano-Gallego L., Alberola J., Ferrer L., Sánchez A., Roura X. (2006). Advantages of Real-Time PCR Assay for Diagnosis and Monitoring of Canine Leishmaniosis. Vet. Parasitol..

[19] Lombardo G., Pennisi M.G., Lupo T., Migliazzo A., Caprì A., Solano-Gallego L. (2012). Detection of *Leishmania infantum* DNA by Real-Time PCR in Canine Oral and Conjunctival Swabs and Comparison with Other Diagnostic Techniques. Vet. Parasitol..

[20] Bento M.F., Souza G.S.D., Vieira B.S., Silva Â.A.D., Krawczak F.D.S., Moura V.M.B.D.D. (2023). Sensitivity of PCR in Conjunctival Swab Samples for the Diagnosis of Canine Visceral Leishmaniasis: A Systematic Review and Meta-Analysis. Rev. Bras. Parasitol. Vet..

[21] Mendoza-Roldan J., Benelli G., Panarese R., Iatta R., Furlanello T., Beugnet F., Zatelli A., Otranto D. (2020). *Leishmania Infantum* and *Dirofilaria immitis* Infections in Italy, 2009–2019: Changing Distribution Patterns. Parasites Vectors.

[22] Oliva G., Bongiorno G., Ayhan N., Kolářová I., Foglia-Manzillo V., Kirstein O.D., Jelínková K., Dvořáková B., Risueño J., Verdú-Serrano E. (2026). Dogs as Sentinel Hosts for Sand-Fly-Borne Infections in the Mediterranean Basin: A Multinational Serological Survey. Parasites Vectors.

[23] Maroli M., Gradoni L., Oliva G., Castagnaro M., Crotti A., Lubas G., Paltrinieri S., Roura X., Zini E., Zatelli A. (2010). Guidelines for Prevention of Leishmaniasis in Dogs. J. Am. Vet. Med. Assoc..

[24] Roura X., Paltrinieri S., Cortadellas O., Fondati A., Lubas G., Zini E., Otranto D., Benali S.L., Zatelli A. (2026). Updated Canine Leishmaniosis Working Group Recommendations for Leishmaniosis in Dogs: Q&A on Clinical Management. Parasites Vectors.

[25] Baxarias M., Álvarez-Fernández A., Martínez-Orellana P., Montserrat-Sangrà S., Ordeix L., Rojas A., Nachum-Biala Y., Baneth G., Solano-Gallego L. (2018). Does Co-Infection with Vector-Borne Pathogens Play a Role in Clinical Canine Leishmaniosis?. Parasites Vectors.

[26] Beasley E.A., Pessôa-Pereira D., Scorza B.M., Petersen C.A. (2021). Epidemiologic, Clinical and Immunological Consequences of Co-Infections during Canine Leishmaniosis. Animals.

